# Clinical presentation and treatment of 2 patients with infection caused by *Chromobacterium violaceum in Vietnam*

**DOI:** 10.1186/s12879-024-09390-1

**Published:** 2024-05-21

**Authors:** Bùi Văn Nam, Bùi Thanh Hà, Đặng Thị Thúy, H. Rogier van Doorn, Bùi Vũ Huy

**Affiliations:** 1 National Hospital for Tropical Diseases, Hanoi, Vietnam; 2 Hanoi University of Medicine and Pharmacy, Hanoi, Vietnam; 3 Oxford University Clinical Research Unit, Hanoi, Vietnam; 4 Hanoi Medical University, Hanoi, Vietnam

**Keywords:** *Chromobacter violaceum*, Pustules, Opportunistic, Antibiotic, Sepsis

## Abstract

*Chromobacterium violaceum* is a rare but severe and often fatal cause of disease in humans. We present 2 clinical cases of sepsis and skin abscesses / cellulitis caused by *C. violaceum* seen in a referral hospital for infectious diseases in Vietnam. Both patients survived, but appropriate antibiotic treatment was only installed after culture of the organism. We reviewed and summarised the characteristics of *C. violaceum* infection and treatment.

## Background

*Chromobacterium violaceum* is a gram-negative, facultatively anaerobic, motile bacillus, belonging to the family of *Neisseriaceae. C. violaceum* is a common environmental bacterium, existing in soil and water. It rarely causes disease in humans [1, 2]; 200 cases of infection caused by *C. violaceum* have been reported in the global literature. The disease occurs in both adults and children and is usually associated with penetrating trauma involving soil or water. Most reported cases are isolated, and the mortality rate is high [2–4]. Limited knowledge about *C. violaceum* infections can present obstacles to the diagnosis and treatment of the disease. Diagnosis of *C. violaceum* cases requires microbiological evidence and susceptibility results, but there are no treatment guidelines or consensus on treatment regimens [4]. As with infections caused by other unexpected microorganisms, delayed diagnosis and treatment with ineffective antibiotics, can lead to worsening and life-threatening illness or death while waiting for microbiological confirmation [2–4].

In Vietnam, *C. violaceum* is an infrequent cause of skin infection and sepsis [5, 6]. The National Hospital for Tropical Diseases (NHTD) is a tertiary referral hospital for infectious diseases in Hanoi, in northern Vietnam. In the hospital's Pediatrics Department, 2 cases of infection caused by *C. violaceum* were seen within a short interval. Diagnosis and appropriate treatment of the disease were delayed, but eventually applied and both patients survived. Here we share our observations and experience in the diagnosis and treatment of these two cases [2, 7].

## Case presentations

### Case 1

Female, 8 years old, BMI 14.2, from Thai Nguyen province. Discharge diagnosis: sepsis, complications of osteomyelitis due to* C. violaceum.*

Medical history: the patient was diagnosed and treated for pulmonary tuberculosis (TB) twice (at 3 and 7 years old) and completed treated with the standard 6-month treatment regimen.

Her diseases started with pustules on the left shoulder, with a size of 1 × 1cm, that were painful and progressively swollen. On the second day of illness, the patient developed a fever of 38.6 ^0^C. On day 3, the pustules spontaneously ruptured, showing cloudy fluid and leaving ulcers, while fever remained at 39 ^0^C. The patient was admitted to the district hospital for 5 days of cephalosporins antibiotic treatment. However, the fever increased (39 – 40 ^0^C) under antibiotic treatment and ulcers on the patient’s shoulder enlarged. The patient was transferred to the provincial hospital. She received treatment with cefoperazole and tobramycin during 5 days. The fever did not improve, blisters appeared all over her body, including on hands and feet, with size from 2mm to 3cm. Over 5–7 days the blisters evolved from a maculopapular rash to pustules which left ulcers after rupture. Simultaneously, there was swelling and pain in the dorsum of the right hand and in both ankles. On day 13 of illness (1 October 2022), the patient was admitted to NHTD, diagnosed with sepsis, suspected of and treated as *Burkholderia pseudomallei* (melioidosis) [4]. Intravenous antibiotic therapy consisting of ceftazidime and linezolid was used. Blood culture results on day 16 showed *C. violaceum*. Based on the antibiogram the intravenous antibiotic therapy was changed to ciprofloxacin and meropenem [4]. The clinical course, laboratory tests, and treatment solutions are presented in Table 1. The condition of the patient worsened with respiratory failure, bleeding at the injection site, swelling of the hands and limbs, and bloating of the abdomen. However, after 10 days of appropriate treatment (October 4^th^ – 14^th^), the general clinical condition improved. An MRI scan of the still swollen right hand on this day showed osteomyelitis.

**Table 1:** Clinical, laboratories and treatment of case 1, female, 8 years old, with septicemia

Time	First hospitalization (Oct 1^st^, 2022 – Dec 13^th^, 2022)					Second hospitalization	Third hospitalization
	**Oct 1**^**st**^	**Oct 6**^**th**^	**Oct 14**^**th**^	**Nov 1**^**st**^	**Dec 1**^**st**^	**Jan 9**^**th**^—**16**^**th**^, **2023**	**Jan 27**^**th**^ – **Feb 17**^**th**,^ **2023**
**Clinical**							
Temperature	39 – 40^0^ C	39^0^ C	38^0^ C	38.2^0^ C	No fever	36.9^0^C	No fever
Glasgow Come Scale	15	15	15	15	15	15	15
Pulse (times/min)	100	150	130	-		86	
Blood pressure (mmHg)	90/60	100/50	-	-		100/60	
Breathing (times/min)	20	50	35	25			
Rale in the lungs	(-)	( +)	( +)	(-)	(-)	(-)	
SpO2	0.98	0.85	0.98	0.98			
Skin	Blister rash, scattered	Cloudy blisters, whole body	Blisters scab	Blisters scab	Blisters scab	Normal	
Other issues	Left shoulder ulcer 1 cm, yellow discharge	Right hand swelling	Swelling of right forearm and back of hand	Cellulitis, fluid leakage in the right hand	Pus-inflamed distal third of the right lower leg	Right heel abscess leaking pus	Right heel abscess without pus leakage
	Swelling of the right hand and ankle joints on both sides	Bleeding at the site of infusion		Many small abscesses on both sides of the knee and left elbow			
		Severe edematous extremities					
		Abdominal distended					
Liver, spleen (below costal margin)	Spleen 2 cm	Enlarged liver and spleen	Normal	Normal	Normal	Normal	
**Laboratory**^**a**^							
Red blood cells (T/L)	4.63	3.67	3.39	3.92	4.59	5.13	5.82
Hb (g/L)	113	92	87	98	109	119	135
Hct	0.34	0.28	0.27	0.3	0.33		
White blood cells (G/L)	19.8	5.7	5.9	11.5	6.2	5.5	5.5
Neutrophil (%)	83.6	75.5	68.1	75.9	49.6	34.4	42.3
Platelet (G/L)	232	12	167	534	336	287	257
CD4 + T cells					346		
CRP (mg/L)	228.3	208.4	121.6	79	23.5		12.6
AST/ALT (U/L)	114/259	106/193	184/170	30/9	33/16	15/32	
Albumin/protein	-	25/56	29			42/85	
Creatinine(mmol/L)	38	37	23	27	35	34	44
Na/K (mmo/L)	132/3.4	135/3.0	131/3.8	139/3.6	134/3.6	137/3.9	137/4.2
PT%		0.75	0.76	0.73			
APTT (s)		35	31.4				
Fibrinogen (g/L)		5.15	4.98				
D-Dimer (ng/ml)		16015	16739	5241			
Blood culture	( +)^b^		(-)	(-)		(-)	
Wound culture	(-)			(-)		(-)	
Urine test (protein; red cells)	0.3 g/L; 200	1.0 g/L; 80	(-)			(-)	
X-Ray	Pneumonia	Pneumonia	Pneumonia	Osteitis of right wrist^c^		Osteitis of right calcaneal	Multiple tunnel-like bone resorption in the right heel
CT/MRI			Pneumonia, stable tuberculosis and osteomyelitis of the right hand^d^		Left tibial osteomyelitis		
**Diagnosis**	Sepsis, complications of osteomyelitis due to *C. Violaceum*					Osteitis of calcaneal	Osteitis of calcaneal
**Treatment**	- The combination of ceftazidime (02–04/10) and linezolid (01 – 11/10)					- Dredge the inflammation	- Inflammatory pulp curettage, ciprofloxacin + ceftriaxon
	- The combination of meropenem and ciprofloxacin (04 /10– 01/12)					Outpatient, oral moxifloxacin for 2 weeks	Outpatient, oral ciprofloxacin for 3 weeks
	Oral combination of levofloxacin and co-trimoxazole (01—13/12)						
	HFNC Flow, platelet transfusion: 150 ml (06/10) and 250 ml (07/10), Human albumin: 20 g (05, 06 and 07/10), red blood cell transfusion: 250 ml (05, 12 and 17/10)						
	The right hand: drainage (01/11) and curettage inflammation (12/11)						
	December 13^th^: discharged from hospital, oral levofloxacin for 28 days, follow-up every month						

^a^Other tests: including HIV test, influenza A, B, Covid -19, nasopharyngeal culture, and blister fluid culture,.doppler echocardiogram, cerebrospinal fluid analysis and brain CT scan. All results were negative

^b^Blood culture results after 3 days identified C. violaceum, sensitive to levofloxacin, ciprofloxacin, imipenem and resistant to Ceftazidime

^c^X-ray taken on November 18^th^

^d^MRI results: Lung scan on October 17^th^, metacarpal scan on October 19^th^

**Fig. 1:**
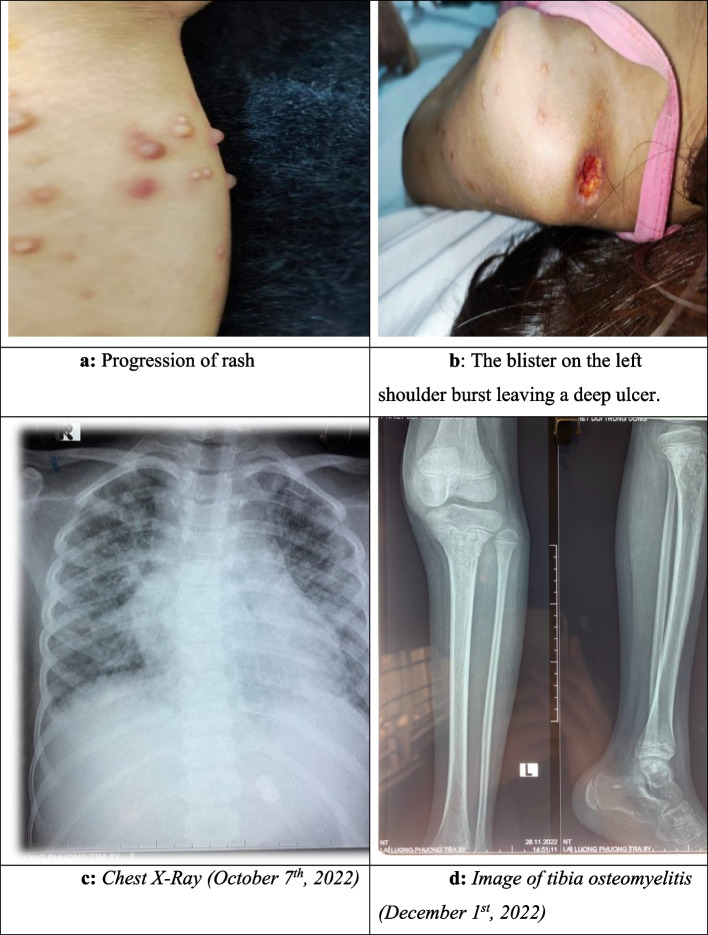
Case 1, female, 8 years old, with sepsis, complications of osteomyelitis caused by *C. violaceum*

After 4 weeks of treatment [4], small abscesses appeared scattered over the knees and lower legs on both sides, and left elbow. Inflammation indices (white blood cell count, neutrophil rate, C-reactive protein) were still increased, although the results of blood cultures and fluid cultures of new abscesses were all negative. Due to fluid leakage from the right hand osteomyelitis, the patient was drained (November 1^st^) and curettage (November 12^th^) of the hand osteomyelitis as directed by the surgeon. After 8 weeks of intravenous ciprofloxacin and meropenem treatment (from October 4^th^ to December 1^st^), clinical manifestations and inflammatory indicators were normal, but there was still an abscess in the lower third of the left leg and MRI showed tibial osteomyelitis. The patient was prescribed oral levofloxacin and co-trimoxazole for 2 weeks (from December 1^st^ to 13^th^). During this time, the pus fistulas dried up, the patient was able to walk, and was discharged from the hospital for outpatient treatment with levofloxacin for 28 days. The patient was readmitted to the hospital twice (January 9 and January 27, 2023), and was diagnosed with osteitis of right calcaneal with pus fistula. The patient had calcaneal osteoarthritis curettage according to the surgeon's instructions. After 5 months of monitoring and treatment (from October 1^st^, 2022 to February 17^th^, 2023) the patient is in stable condition, with no sequelae (Fig. 1).

### Case 2

Female patient, 13 years old, BMI: 19, from Bac Giang province. Discharge diagnosis: skin abscess and cellulitis due to* C. violaceum.*

Medical history: The patient was diagnosed with pulmonary tuberculosis at 2 months old, idiopathic thrombocytopenic purpura at 8 years old, and both conditions have been treated successfully. The patient has never been vaccinated against CoV-19 and in August 2022 was diagnosed with CoV-19 with respiratory failure at NHTD. In this episode, the patient developed symptoms of continuous fever, peaking at 40 ^0^C and body aches. By the third day, blisters appeared all over the body, but did not form ulcers and swelling and pain in the right shoulder and elbow joints. The patient was admitted to the National Lung Hospital and was treated with cefepime and amikacin for 3 days, but the disease did not improve. Treatment was then changed to linezolid and meropenem for the next 3 days. However, the patient still had a continuous high fever, and pain in the shoulder and right elbow, with swelling of the ankle joints on both sides. She was transferred to NHTD.

**Fig. 2:**
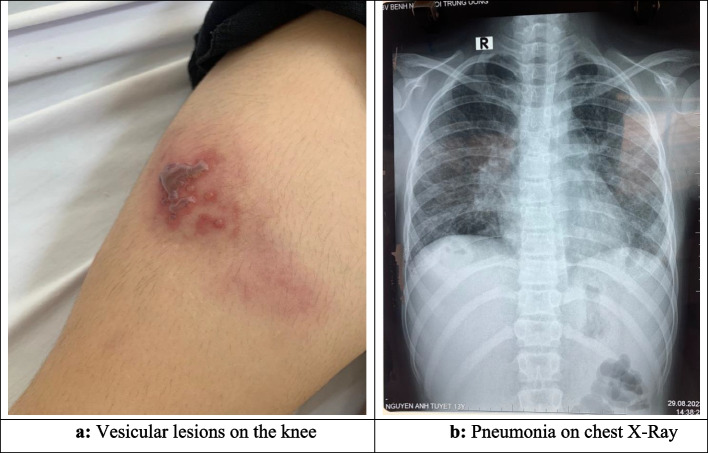
Case 2, female, 13 years old with skin abscess and cellulitis caused by *C. violaceum*

The patient was admitted to NHTD on day 8 of illness (August 28^th^, 2023), with a fever of 40 ^0^C, scattered clear blisters on the skin, swelling and pain in ankle and knee joints on both sides, and shoulder and elbow joint on the right side only (see Table 2). Laboratory results showed elevated inflammatory indices (white blood cell count 19.4 G/L, neutrophil ratio 71.4% and CRP 107.9 mg/L). The patient was diagnosed with skin abscesses and cellulitis and was treated with an intravenous regimen of cefepime and vancomycin. Culture of blister fluid showed *C. violaceum* and antibiotic therapy was switched to ciprofloxacin plus cefepime. The disease condition improved quickly and after 5 days, the fever was gone, the blisters on the skin gradually dried, but the joints were still swollen, painful and inflammation indexes were still high (white blood cell count 14.8 G/L, CRP 39.9 mg/L). After 2 weeks of treatment (from August 30^th^ to September 15^th^), the blisters were dry, scaly, the joints were no longer swollen, and inflammation indicators were normal. The patient continued to receive antibiotics and was clinically monitored for 4 weeks [4], her condition remained stable and she recovered with no complications, and was discharged from the hospital. The results of the monthly re-examination showed that the disease was completely cured (Fig. 2).

**Table 2:** Clinical, laboratory, treatment of case 2, female, 13 years old, with skin abscess and cellulitis

Time	Aug 28^th^, 2023	Sep 3^rd^, 2023	Sep 15^th^, 2023	Sep 28^th^, 2023
**Clinical**				
Temperature	40^0^C	36.5^o^C	36.5^o^C	36.8^o^C
Glasgow Come Scale	15	15	15	15
Pulse (times/min)	80			
Blood pressure	100/60 mmHg			
Breathing (times/min)	20			
Rale in the lungs	(-)	(-)	(-)	(-)
Skin	Blister rash, scattered	Blisters scab	Dry, peeling blisters	Normal
Other issues	Swollen and painful ankle joints, knee joints on both sides, and shoulder joints, elbow joints on the right	Ankle joints, knee joint on both sides and the right shoulder joint were still swollen	Normal	Normal
Liver, spleen (below costal margin)	(-)	(-)	(-)	(-)
**Laboratory**^**a**^				
Red blood cells (T/L)	3.79	3.71	4.48	
Hb (g/L)	96	94	113	
Hct	0.285	0.278	0.337	
White blood cells (G/L)	19.4	14.8	6.9	
Neutrophil (%)	71.4	67.7	50.9	
Platelet (G/L)	186	577	257	
CRP (mg/L)	107.9	39.9	13.8	
AST/ALT (U/L)	15/18		19/11	
Creatinine (mmol/L)	30		29	
Na/K (mmol/L)	139/3.0		134/3.6	
Blood culture	Bacteria (-), TB (-)			
Blister fluid				
AFB staining	(-)			
*PCR TB*	(-)			
Culture	*C. violaceum* ( +)^b^			
Chest X-Ray	Pneumonia			
Abdominal ultrasound	Enlarged liver			
Joint ultrasound	Right knee joint effusion, right shoulder joint muscle tendon echo reduction			
**Treatment**	Vancomycin 55 mg/kg/day combined with cefepime 150 mg/kg/day (August 28^th^ – 29^th^)			
	Ciprofloxacin 25 mg/kg/day combined with cefepime 150 mg/kg/day (August 30^th^—September 28^th^)			

^a^Other tests, including HIV rapid test (-), echocardiogram and cerebrospinal fluid test were all normal

^b^On the antibiogram, the bacteria were sensitive to fluoroquinolones, aminoglycosides, carbapenems

## Discussion

In Vietnam, infectious diseases are still having a strong impact on human health. Changes in climate, land use and demographics may affect risk of exposure to potential pathogens including in the environment and among animals [2, 8].

*C. violaceum* has been recognized as a bacterium that can cause human infections, including opportunistic infections, as it is commonly found in people with immunodeficiency or comorbidities [2, 4]. Infections caused by *C. violaceum* have been reported sporadically globally, but were concentrated in Southeast Asia [4]. *C. violaceum* can cause a variety of diseases, including urinary tract infections, gastro-intestinal infections, osteomyelitis, meningitis, and sepsis. The estimated fatality rate is high at 53% [2] to 60% [3, 4]. Clinically, diseases caused by *C. violaceum* can be confused with other diseases, especially those caused by *B. pseudomallei* (melioidosis), and diagnosis needs to be based on blood culture results or fluid from infection foci [2, 4]. *C. violaceum* has been reported to be sensitive to fluoroquinolones, chloramphenicol, tetracycline, co-trimoxazole, imipenem and aminoglycosides and resistant or less susceptible to cephalosporins and colistin [3, 4]. However, due to the disease's rarity and quick progression, diagnosis is often delayed as is appropriate antibiotic treatment. Currently, no specific treatment guidelines are available [1, 2]. Furthermore, similar as in melioidosis, the disease may relapse after 2 weeks of appropriate treatment and oral co-trimoxazole, doxycycline, or ciprofloxacin have been suggested for use for several weeks to months to prevent recurrence [2, 4].

In our two cases, regarding diagnosis, case 1 was diagnosed with sepsis [9] with complications of osteomyelitis caused by *C. violaceum* (see Table 1) and case 2 was diagnosed with skin abscess and cellulitis caused by *C. violaceum* (see Table 2). The MaldiTOF method was used to identify pathogenic bacteria. Both of these cases had a history of tuberculosis, managed by the tuberculosis prevention system in Vietnam, so we suspected these individuals had an immunodeficiency condition [10]. In the first patient, after appropriate treatment, the disease stabilized. Regarding the route of entry of bacteria, in both patients, we suspected the route of entry was the skin [3]. In case 1, pustules on the skin appeared one day before fever, while in case 2, although the route of bacterial entry remains unclear, this patient only had blisters and ulcers on the skin, followed by joint involvement. In case 1 culture results identified *Chromobacterium violecium* in the blood, while in case 2 the bacteria were only detected in the pustules. In our opinion, either the pustules were a symptom of *C. violaceum* sepsis, or the pustules were a presentation of another undiagnosed illness and *C. violaceum* was environmentally introduced and then caused secondary sepsis. Given her high fever and response to the antibiotics we treated, the first hypothesis seems more reasonable.

Clinically, the two patients had two main characteristics: 1/infection syndrome (fever and increased inflammatory indexes) and 2/cellulitis and infectious large arthritis (broken blisters leaving deep ulcers, swelling and inflammation of knees, shoulders, elbow joints). In addition, there was pneumonia on chest X-Ray. These characteristics are similar to melioidosis, a disease that is endemic in Vietnam [11]. Although the diagnosis of *C. violaceum* or *B. pseudomallei* bacteria must be based on blood/pus culture [2, 4], both of our cases had inflammation of the large joints, while arthritis is not common in melioidosis [12]. Simultaneously with the bacterial culture results, antibiograms were performed on both cases. For case 1, bacteria were sensitive to levofloxacin, ciprofloxacin, imipenem and resistant to ceftazidime. In which the MIC tests for levofloxacin, ciprofloxacin, imipenem and ceftazidime were 0.002 µg/mL, 0.004 µg/mL, 2 µg/mL, 256 µg/mL respectively (all using the ETEST method). For case 2, on the antibiogram, the bacteria were sensitive to ciprofloxacin, levofloxacin, amikacin, gentamincin, meronem. The MIC test for levofloxacin and meropenem were 0.003 µg/mL and 0.25 µg/mL, respectively (ETEST method). For the remaining 3 antibiotics, the DISK method showed qualitative results and the bacteria were sensitive to these antibiotics.

Regarding treatment: We recorded that ciprofloxacin was effective in both cases, based on the antibiogram and treatment results [2]. In our opinion, although case 2 was treated in combination with cefepime, the antibiogram results in case 1 (limited antibiogram) showed that *C. violaceum* was resistant to ceftazidime. Third generation cephalosporins are known to not be effective for *C. violaceum* [2, 4, 5]. While case 1 was treated with a combination of two antibiotics effective for *C. violaceum* according to the antibiogram, the treatment was initiated late (day 16 of the disease), allowing the disease to progress further. The patient had signs of septic shock (rapid pulse, increased blood pressure, respiratory failure) and complications of osteomyelitis (metacarpal, tibia, calcaneus). The consequences led to a prolonged treatment period and the need to be readmitted to the hospital twice for calcaneus curettage intervention.

Thus, treatment of *C. violaceum* should use fluoroquinolone and carbapenems can be used as an alternative antibiotic [13]. In cases where it cannot be distinguished from *B. pseudomallei*, carbapenems should be used, as they are effective against both types of bacteria, while fluoroquinolones are not recommended in the treatment of *B. pseudomallei* [12]. Although the bacteria were susceptible to aminoglycosides on antibiograms, in our opinion, infections due to *C. violaceum* are serious and occur in immunocompromised individuals. This will require a long course of antibiotics, so aminoglycosides should only be used as a combination antibiotic treatment.

Regarding treatment duration, in case 2 (see Table 2), after 2 weeks of treatment, clinical manifestations and inflammatory indices had returned to normal [13, 14]. Based on available guidance from literature [2, 4, 15], the patient continued to receive antibiotic treatment and was monitored for a full 4 weeks. During this period, there were no further developments. The results of the monthly follow-up examination showed that the patient was healthy and had resumed normal daily activities. However, in case 1, after 2 weeks of combining ciprofloxacin and meropenem, the patient remained febrile and otherwise symptomatic (signs of pneumonia, swollen and inflamed joints, CRP increased) and treatment was continued. After 4 weeks of treatment (November 4^th^, 2022), the patient still had fever, inflammation and fluid production from the hand joints, small abscesses appearing on both knees and elbows, inflammatory indices increased. After 8 weeks of treatment, the patient’s condition and inflammatory indices had returned to normal, although there was tibial osteomyelitis. During the next 3 months of follow-up (December 1^st^, 2022—February 2023), in addition to the treatment of osteomyelitis according to surgical indications (using ciprofloxacin and curettage of the calcaneus twice), all symptoms had disappeared. In our opinion, based on these 2 cases, the duration of intravenous antibiotic treatment should be at least 2 weeks [13, 14], and can be continued for 8 weeks based on patient's condition. After this period, treatment can be used to prevent disease recurrence with oral antibiotics, depending on the patient's condition and nature of specific complications.

## Conclusion

We have reported two cases of infection with *C. violaceum*, in two patients who likely had some form of immune-deficiency. Infections presented as an infectious syndrome with signs of penetrative skin trauma in one. Diagnosis and treatment were delayed and antibiogram based treatment was continued until symptoms resolved. *C. violaceum* should be considered in infectious syndromes in endemic countries when cephalosporins have no effect. These case reports also emphasize the importance of taking blood and site cultures to aid in diagnosis and appropriate treatment.

## Data Availability

No datasets were generated or analysed during the current study.

## Abbreviations

NHTD: National Hospital for Tropical Diseases
BMI: Body Mass Index
TB: Tuberculosis
CRP: C-reactive protein
MRI: Magnetic Resonance Imaging
